# Thinking with Civets: The Role of Zoos in the Decolonisation of Animal Tourism

**DOI:** 10.3390/ani13111739

**Published:** 2023-05-24

**Authors:** Jes Hooper

**Affiliations:** The Department of Social and Political Sciences, Philosophy, and Anthropology, The University of Exeter, Exeter EX1 2LP, UK; jh1220@exeter.ac.uk

**Keywords:** *Viverridae*, zoos, animal welfare, conservation, decolonisation, animal tourism

## Abstract

**Simple Summary:**

The conditions afforded to zoo animals across the globe remain diverse and disparate. This paper brings two forms of animal commodification into dialogue: (1) the management of civets in global zoos and (2) the rising trend in civet coffee production and tourism in Asia. By qualitatively analysing the entanglements between colonialism, animal welfare, and conservation in civet tourism, this paper calls for enhanced reflexivity and decolonisation of animal-based tourism. Suggestions are made on how zoos may answer this call.

**Abstract:**

Civets belong to the family *Viverridae*, an ancient line of ‘cat-like’ animals. Despite their large geographic distribution across southeast Asia and parts of Africa, little scientific attention has been attributed to *Viverrids* or *Viverrid*–human relations. This paper applies the lens of civets to explore the tensive intersection between animal welfare, conservation, and colonialism within the tourism landscape. Through thinking with civets, this paper brings two forms of animal commodification into dialogue: (1) the management of civets in zoos around the globe and (2) the rising trend in civet coffee production and tourism in Asia. By qualitatively analysing the entanglements between colonialism, animal welfare, and conservation and how each impacts the lives and treatment of civets in tourism, this paper calls for enhanced reflexivity and thus the decolonisation of animal-based tourism. Suggestions are made on how zoos may progress towards the decolonisation of animal tourism, and the argument is made that zoos are well positioned and morally obligated to answer this call. By doing so, greater attention can be given to the animals whose lives are most affected by the global tourism landscape.

## 1. Introduction

I will always remember the first time I encountered a member of the *Viverridae* family. As an eager undergraduate student studying Animal Science, I would frequently volunteer at my nearest accredited zoo. A small, family-run business, the zoo had a modest collection of exotic and domestic species. With a young primary target demographic, there was much emphasis on children’s education. Fun facts were painted on signs throughout the animal collection, and children were encouraged to take part in ‘Zoolympics’—activity stations placed intermittently throughout the zoo trail where children and their guardians could see if they could run as fast as a cheetah or jump as far as a kangaroo. Of the animal displays, there was an overrepresentation of round-faced and fluffy species, from domestic rabbits to red pandas—those with charismatic features that zoo visitors consider most appealing [1].

As I arrived for my shift on this particular day in 2011, the mood in the zoo felt different. There was an excited murmuration of volunteer gossip filling the air as rumours abounded about the latest zoo arrival—today was the day that the new binturong (*Arctictis binturong*) exhibit opened to the public. Having never heard of this species before, I eagerly awaited my opportunity to go and see them. As soon as it was lunch break, I diverted away from the direction of the food stalls and headed to the new binturong enclosure. Whatever image of an animal I could have conjured would certainly have been less unusual than that which met me upon my arrival. I considered the animals who looked back at me nothing short of captivating. Weighing up to 20 kg and measuring ~1.8 m from head to tail, with large paws, long shaggy grey and black fur, and elliptical pupils, binturong also possesses a powerful prehensile tail for arboreal locomotion [2]. Known affectionately as ‘bearcats’ given their size and anatomically cat- and bear-like features, binturong are an ideal species to engage zoo visitors [3].

Their large size and preference for height make binturong visually noticeable. Despite their crepuscular nature, binturong move around their enclosure and rest in full view of zoo visitors, and their activity patterns are unaffected by the presence or noise of spectators [4]. Their lack of direct relatedness to bears or cats makes for fun facts for enclosure signage, and their musk secretions give off a distinct pop-corn smell [5] for a multisensorial immersive zoo experience. Smell is an important sense for forming memories and arousing feelings [6], so odour may offer zoo education programmes an additional factor to contribute to long-lasting educational messages. Binturong are also formally recognised as in need of conservation, listed as ‘Vulnerable to Extinction’ (VU) on the IUCN Red List of Threatened Species [7], another characteristic that is favoured by zoo visitors [8]. On reflection, it was unsurprising that binturong were imbued with exoticism and mystery to me, for despite having lived alongside humans throughout history, their illusive nature had left binturong and their civet (*Viverrid*) relatives as an understudied, albeit ancient, set of species [9]. The inclusion of *Viverrids* in zoos could also provide an opportunity to enhance scientific knowledge. In all, binturong make for a sensible species selection for zoos, as curators increasingly attempt to fulfil a variety of (often conflicting) institutional objectives.

Most accredited zoos cite their primary objectives as conservation, education, recreation, and research [10], though academics have often debated the ability of zoos to fully meet these objectives [11,12,13]. Some scholars have condemned the keeping of endangered animals captive as nothing more than a “macabre” exercise of human curiosity [14], whereas others have noted the benefits of zoos as spaces for fostering pro-conservation behaviour [15] and contributing to the preservation of declining species [16]. Within anthropology, the zoo is notable for its geographical and theoretical positioning alongside museums [17,18], both of which have emerged as places where specimens are displayed in pursuit of human knowledge [19,20,21], see the zoo not as a space for animals but as human-constructed cultural institutions that disseminate social and cultural meaning through the display of live animals ([20,21] are translated to English in [22]). This article is grounded in a similar anthropological perspective. I am concerned both with the history and the future possibilities of zoos and their role within contemporary global animal tourism. I am especially interested in the tenacious intersection between animal welfare, conservation, and colonialism within the tourism landscape.

The way zoos are represented in different cultural contexts and how accredited zoos in economically advanced nations position themselves on the global stage is an area of research that has gone relatively understudied, as is the link between colonial narratives and animal welfare within tourism. As I will go on to explain in the forthcoming sections, the role of zoos throughout Europe in the 19th to 21st centuries has shaped and responded to public perceptions of and concerns for animal welfare in tourism. However, as I will demonstrate, there are many ways that accredited zoos fail to adequately address animal welfare in the global tourism and leisure space, in which they are themselves firmly situated. Nor do they always reflect upon their colonial histories when collection planning or offering recompense to economically emerging nations within endangered species home ranges. While zoos have evolved substantially from their curiosity-driven emergence to their current position as leaders in conservation, education, animal welfare, and research, it was colonialism that made the modern zoo’s emergence possible [23]. It is with the lingering shadow of the zoos’ colonial origins that this article is concerned, as I ask for further reflection on how zoos may continue to move beyond their colonial past. Just as [24] advocated for the decolonisation of tourism literature, this essay advocates for the decolonisation of animal tourism and argues zoos are morally and situationally well positioned to pursue this mission.

This essay contributes to the growing anthropological literature concerned with the ‘species turn’, the refocusing of scholarly inquiry towards the role of animals within human-dominated societies [25]. It is part of an ongoing multi-year investigation into human–civet relationships that arguably became embedded in my consciousness during my first encounter with a binturong. For the past four years, I have been conducting a multi-sited and multi-method approach to the anthrozoological investigation, a critical analysis of contemporary human–civet relationships. This article is a qualitative literature review that has formed alongside the collection of empirical data, which is presented elsewhere in the literature (see [9,26,27]). Through thinking with civets in this essay, I first address the colonial underpinnings of zoos, their advances towards improved standards of animal welfare, and the demographics of *Viverrid* species housed in the zoos. I then introduce the colonial origins of civet coffee and the emergence of civet coffee tourism before discussing the commonly associated animal welfare and conservation issues and the ways zoos are used by tourists to condone and condemn poor animal welfare practises. Finally, I conclude the article with a series of recommendations that accredited zoos could strive towards in a bid to decolonise global animal tourism. While some of the recommendations I make are theoretical and thus somewhat limited in their ability to be directly implemented, I hope that this essay provokes conversation and inspires accredited zoos from economically advanced nations to adopt greater reflexivity in their current and future practises.

## 2. Zoos: Past and Present

The colonial histories of zoos are well documented in anthropological and historical texts [23,28,29]. While many of the first living animal specimens entered Europe via the slave trade routes of the 1770s [30], by the 1900s, humans were also subject to zoo display as a spectacle of the primitive [31]. Certainly, entertainment and a desire to satisfy human curiosity about the exotic were prime directives of early public zoos [28]. Enclosures were built with panoptic displays designed for unobstructed onlooker observation [32], and visitors could ride on the backs of elephants or watch chimpanzees perform tea parties and drink alcohol [33]. It was in the 1950s that zoo animal welfare began to take centre stage as zoos (particularly those in Europe and North America) were pressured by the general public to improve captive conditions for the animal’s experience [34]. Such a shift came in the wake of increased accessibility to television and the popular broadcasting of nature documentaries, which showcased for the first time the natural habitats from which zoo animals had been separated. Sir David Attenborough’s television series ‘Zoo Quest’, for example, transported UK viewers to the wild to see zoo animals in their “proper setting” [35]. The imagery of wild animals immersed in natural landscapes and expressing active species-typical behaviour such as play, allogrooming, hunting, and foraging inadvertently lead viewers to question the appropriateness of zoo environments. Thus, it is notable how animal welfare and ethics are entangled. Where animal welfare is used to describe an animal’s psychological and physical health [36], where their ability to cope with their environment can be measured by parameters such as intrinsic need [37], it was in questioning the ethics of whether animals were deserving of having these needs met that animal welfare became central within the zoo industry. Therefore, from the mid-20th century, naturalistic and immersive enclosures were more systematically adopted in zoos in economically advanced nations, along with a renewed commitment to prove to the public that the zoo as an institution remained a valuable societal asset for both humans and animals [10].

Today, the global modern zoo industry attracts more than 700 million annual visitors [38], and visitor satisfaction continues to be of paramount importance. Zoo visitors not only claim to rate animal welfare as a primary concern when visiting zoo collections [39], but zoo visitors are said to learn more [40], view zoos more positively [41], and commit to pro-conservation behaviour more readily [42] when they have witnessed zoo animals performing natural and desirable behaviour. Similarly, the viewing of animals expressing inactivity or stereotypic behaviour can negatively impact the experience of zoo visitors [43]. When animal welfare negatively shapes the zoo visitor’s experience, this in turn risks the zoos’ financial security and their ability to meet their education and conservation objectives.

The formation of global and regional accreditation programmes, such as the World Association of Zoos and Aquariums (WAZA), European Association of Zoos and Aquariums (EAZA), Association of Zoos and Aquariums (AZA), British and Irish Association of Zoos and Aquariums (BIAZA), and South East Asian Zoo Association (SEAZA), to name a few, have each emerged in response to public demand to hold zoos accountable to higher standards than are required by law in terms of institutional commitments to animal welfare, conservation, and visitor education. The accrediting bodies advocate for a holistic approach to animal welfare provisions and assessments. The WAZA defines animal welfare, for example, as “a state that is specific for every individual animal; it is how the animal experiences its world and life through its association with pleasant experiences specific for that species such as vitality, affection, safety, and excitement, or unpleasant experiences such as pain, hunger, fear, boredom, loneliness, and frustration” (WAZA, 2023). Accredited zoos are, therefore, guided by comprehensive animal welfare policies, which describe the relationships between animal health, nutrition, behaviour, mental state, and environment. Zoos are also required to assess and monitor the welfare of their animals based on these multiple indicators (see [44,45] for examples). However, animal welfare comes at a high economic price, as does ex situ conservation. Thus, the conditions afforded to zoo animals across the globe remain diverse and disparate. Where high welfare standards, including veterinary care, naturalistic enclosure design, and species-specific nutrition, are often synonymous with accredited and legislated collections, such standards are more commonplace in economically advanced nations [46]. Zoos in the United States alone spend upwards of $1 billion per year on operational costs such as animal food, veterinary care, and enclosure maintenance [47]. Artificial insemination, gamete or living animal transfer, and pre- and post-natal care take knowledge, time, and monetary resources, as does the loan of breeding animals of high conservation and political value (see [48] for giant pandas).

Through the lens of captive breeding programmes, we can also see the entanglement between animal welfare, ethics, economics, and conservation. Breeding is not only paramount for conservation, but baby animals also make zoos more attractive to paying visitors [8]. However, zoo animals are bred in captivity for a variety of reasons, from the maintenance of genetic diversity in assurance populations to providing optimal stocking densities to promote natural social behaviours [49]. While some breeding programmes facilitate conservation via species reintroduction [50,51] others are conducted to supply animals for surrogacy and mentorship purposes [52,53]. Thus, the drivers behind captive breeding can be unique and not always obvious to the visiting public. Breeding success can also be impacted by a range of factors. Mate choice [54], individual personality traits [55], environmental parameters [56], social groupings and reproductive ageing [57], and birth origin [58] are just some of the factors that can impact mate success. Additionally, many of these factors can be present for newly acquired species where species-specific knowledge is lacking and so trial-and-error practises are required [50]. Although the level of the conservation value of captive breeding can differ between programmes, animal welfare is integral to captive breeding regardless of the programme’s purpose. Ref. [59] goes as far as to describe the lack of mate success as a form of “reproductive strike”, a form of resistance to inadequate captive management. Indeed, if a breeding animals’ welfare is compromised, then the likelihood of its breeding can also be cast into doubt [60]. However, irrespective of its importance, it remains puzzling that animal welfare is largely absent from accredited zoo mission statements [10]—perhaps its absence is in recognition of the way zoo objectives can and often do conflict in practise [61].

As an example, zoo visitors are said to be most positively impacted by their feelings of connection with zoo animals [15]. This has, in turn, resulted in the continuation of animal performances and intimate human-animal encounters such as animal feeding or ‘keeper for the day’ experiences. Ref. [62] found that of 1241 surveyed zoo facilities, 75% of accredited zoos offered animal-visitor experiences. All in all, animal-visitor activities were most popular in advanced economic regions, as 90% of North American zoos and 75% of European zoos offered some form of animal-visitor interaction beyond passive observation. Rates were lowest in South America, where 50% of accredited zoos offered animal-visitor experiences. Of all the recorded animal-visitor experiences on offer, 43% of accredited zoos across the world advertised hands-on petting experiences, 28% offered non-hand-feeding interactions, and 23% offered direct hand-feeding opportunities [62]. Such activities have led to concerns that close-contact human-animal interactions present mixed messages to zoo visitors and could therefore promote unethical, low-welfare interactions beyond the zoo visit [63]. Amongst these concerns is that the performative display of exotic species serves as an extension of the zoological industries’ colonial histories, whereby animals serve as entertainers under the guise of educational opportunities [14]. Furthermore, although accredited zoos can house confiscated, abandoned, or rescued animals, including local native wildlife [64], the species selection in zoos is still predominately tied to entertainment-driven colonial interests. Zoos in economically advanced countries continue to prioritise the housing of charismatic endangered species, the two characteristics that zoo visitors value most [1].

Through zoo visitation and portrayals in popular media, charismatic endangered species have, however, been found to inspire pro-conservation behaviour [65]. The role of ambassador animals has been widely discussed in the literature, with concepts such as ‘flagship’ or ‘umbrella’ species undergoing several revisions. Such terms often describe animal species that can serve multiple conservation-education outcomes [66,67,68]. Indeed, [68] suggest that “a species’ potential usefulness as a flagship for conservation could be given added value by its ability to act as a marketing surrogate for a large number of high-priority species.” Where most ambassador species are endangered, [69] proposes the use of non-endangered ‘proxy species’ to represent endangered sister taxa with whom they share a physical resemblance. In a similar line of thought, later in his paper, an argument will be made that neither the ambassador nor its benefactor need be restricted to species formally recognised as endangered. Not only are many non-endangered taxa already in steady decline, but ambassador species could promote good animal welfare practises as well as conservation messages. First, I turn our attention back to the *Viverridae* family as a case study of population and taxonomic trends in species representation in the global zoo industry.

## 3. Civets in Zoos

As demonstrated in Table 1, the most popular *Viverrids* housed in zoos across the world are binturong, and approximately 60% of the world’s captive binturong population is housed in zoos within advanced economies rather than in zoos within the species’ natural range. (The data presented in Table 1 were grouped into Emerging and Advanced Economies as defined per country by the International Monetary Fund (see https://www.imf.org/en/Publications/WEO/weo-database/2023/April/select-country-group, accessed on 25 April 2023)) Other *Viverrid* species of conservation concern (listed as either vulnerable, ‘VU’, or endangered, ‘EN’, on the IUCN) include the Banded palm civet (*Hemingalus derbyanus*), Golden palm civet (*Paradoxurus zeylonesis*), Large-spotted civet (*Viverra magaspila*), and Owston’s civet (*Chrotogale owstoni*). Only Owston’s civet and Banded civet are housed in zoos in economically advanced economies and far fewer numbers than binturong. One contributing factor could be heightened rates of reproductive success for binturong, yet this raises questions as to the strategic value of captive breeding for conservation purposes when conducted so far from a species’ natural habitat. While conservation concern is a likely factor under consideration when collection planning, the binturong’s large charismatic appearance and crepuscular nature are more likely to contribute to their heightened representation within the zoo industry when compared to related species. However, the question remains as to how effective binturong are as zoo ambassadors. How well do binturong in zoos represent the welfare and conservation interests of their wild counterparts and *Viverrid* relatives?

The effectiveness of zoo ambassadors has been long debated in terms of meaningful education [71,72]. Ref. [3], however, argue that binturong are valuable species ambassadors, stating that their inclusion in accredited zoos across the world has helped to generate US $67,520 towards in situ conservation efforts. (The monetary total has been converted here from Euros to US dollars for consistency.) Indeed, ambassador species are often effective in inspiring the public to contribute financially towards conservation initiatives [68], and accredited zoos are an important societal resource for collating conservation funding. The WAZA-accredited zoos, for example, are said to collectively contribute an estimated US $350 million towards wildlife conservation each year [73,74]. In 2021, the AZA-accredited zoos reported spending over $25 million across 30 endangered species as part of the AZA ‘Saving Animals from Extinction’ (SAFE) programme. Its members also reported spending a combined $216.9 million on field conservation programmes in the same year, to the benefit of 954 species [75]. Thus, accredited zoos report a significant contribution towards global ex situ and in situ conservation efforts. According to [76], in the most recent peer-reviewed research investigating the regional contributions of zoos towards conservation, it was zoos and aquariums in North America and Europe that “spent the most by far on wildlife conservation (97% of expenses reported)”. Thus, on this basis, it could be argued that housing endangered and charismatic species in zoos in advanced economies is a strategic benefit to global conservation. However, in reaching the sum of zoos’ conservation contributions, zoo accreditation bodies rely on zoos to self-report their spending, as did Gussett and Dick, who defined wildlife conservation broadly as “in situ conservation of wild species and habitats, including related ex situ work”. By this definition, zoos that reinvested in their captive facilities could claim that doing so was a form of wildlife conservation expense. Furthermore, their research highlighted that zoos in Asia received the greatest numbers of zoo visitors than either North America or Europe. It would be useful, therefore, to know how much zoos in economically advanced regions work with zoos in economically emerging ones and how much consideration visitor demographics are given when collection planning. Conservation education may be better served by targeting the demographics that have greater power to affect positive change for animals by presenting species that visitors have a greater likelihood of engaging with outside of the zoo. One of the biggest threats facing all civet species, for example, is the civet coffee production and tourism industry. Given that consumers from economically advanced nations contribute to global demand [77], the conservation and animal welfare implications of civet coffee ought to be a focal message for *Viverrid* holding collections, and yet very few species most impacted by civet coffee (such as the common palm civet, *Paradoxurus hermaphroditus*) are represented in zoological collections in regions contributing towards civet coffee consumer demand. Furthermore, preliminary research shows that few European zoos that house binturong include civet coffee as an educational message in their collections (Hooper, *unpublished thesis*), despite ethical consumerism being of interest to zoo visitors [78].

As demonstrated in Table 1, common palm civets are notable for their poor representation within zoos in advanced economies. Although perhaps one of the less aesthetically charismatic *Viverrids*, the common palm civet would be well suited to zoo representation. Common palm civets reproduce well in captivity and so would not require the captive population to be supplemented with wild animals to maintain genetic diversity and desired zoo stocking densities [27], and they can adapt well to captive settings when high welfare standards are upheld [79]. As the most used species in civet coffee production and tourism, their absence is a missed opportunity for zoo education. Thus, it may be of little surprise that tourists who visit *Viverrid* range countries are often unaware of the civet species they are most likely to come into contact with, nor are they aware of the welfare or conservation issues associated with civet coffee production [26]. As I will now demonstrate, zoos are already enmeshed in the political and tricky intersection between animal welfare, conservation, and ethics in global animal tourism, and so responding to these issues could be seen as a moral imperative.

## 4. Civet Coffee

Civet coffee is a luxury coffee produced through the digestive tract of the common palm civet and is said to be less bitter and smoother in taste than non-digested varieties [80]. Civet coffee has secured a lucrative commodity value through pervasive marketing claims of the product’s rarity and unique production method, and its origin story has become highly fetishized throughout high-income nations despite the product itself being steeped in colonial heritage [9]. Civet coffee, also referred to in Indonesian as ‘kopi luwak’, is said to have been discovered in Indonesia during the time of Dutch colonial rule at the end of the 16th century [81]. Indonesian farmers were instructed by Dutch colonisers to grow and harvest coffee, though they were prohibited from consuming it themselves. As the story goes, coffee farmers observed that wild civets would enter the coffee plantations at night, where they would select only the ripest coffee cherries for consumption. The only trace of these elusive animals left behind were their faecal droppings, which were studded with partially digested coffee cherries. Forbidden to sample from their own coffee harvest and curious about the caffeinated beverage, coffee farmers turned to the civet faeces upon which the beans were removed before cleaning, drying, and roasting them [82]. Upon recognising this local phenomenon, the Dutch rulers are said to have noted the coffee’s seemingly enhanced taste characteristics [83].

Rising to international fame in the early 2000s after it was featured in a series of Hollywood films and prime-time television programmes in Europe and North America [26], civet coffee’s novel production method, romanticised origin story, and proclaimed rarity saw consumer demand rise exponentially [84]. Whether contemporary civet coffee is a product of authenticity, however, is debatable. Early studies confirmed that civet coffee possessed unique structural and chemical characteristics, said to be the work of the civets’ digestive enzymes [80]. Indeed, civet digestion has evolved to facilitate a varied meso-carnivore diet [82]. More recent research, however, has challenged this analysis and identified flaws in the method of the original research [9,85]. Civet coffee collection methods are also widely falsified [86], and although consumers are led to believe civet coffee is produced through the collection of civet faeces by Indonesian farmers, the reality is the mass farming of caged civets to meet the demand of the global market [87]. Civet coffee’s global market is currently increasing and is expected to reach US $11.75 billion by 2030 [77].

### 4.1. Civet Coffee and Civet Welfare

As a common species without conservation protections, common palm civets are captured from the wild to supply the civet coffee industry. While the civet coffee phenomenon originates in Indonesia, its global market growth has seen the emergence of civet coffee farms across the species’ distribution, including Indonesia, Singapore, Vietnam, and India [77]. The capture of wild civets can take the form of caged capture or snaring, and so civets can often be inflicted with injuries through the process of wild harvesting [86]. Although civet coffee farming is permitted by law in several range countries, research has shown that civet coffee farms are often over the legal stocking density [86] or include endangered civet species [88,89].

On civet coffee farms, civets are housed in rows of stacked cages, usually with one animal per cage, with each cage typically no larger than a small domestic dog crate consisting of slatted or mesh floors designed for ease of faecal collection [27]. Civet welfare is highly compromised in these conditions, as civets are forced to sleep and stand on a wire [90], conditions well associated with injury and abrasions [91]. Through such confinement, civets are also forced to remain close to each other, despite members of the *Viverrid* family expressing a preference for solitary behaviours [92]. Thus, the smell, sound, and sight of other animals can cause significant psychological distress, as civets are unable to hide or increase their distance from caged neighbours. Cage-related injuries such as abrasions to paws and faces and stereotypic behaviour, including pacing and self-mutilation, are all common occurrences [26,86,90].

To obtain the most productivity from the farm practise, farmers report feeding their civets a diet either mostly or exclusively comprised of coffee cherries, and captive civets often die from lack of nutrition and caffeine toxicity [93]. Rather than seeking veterinary care for sick individuals, farmers have been known to release civets into the wild once they become ill, only for the same civet to be recaptured upon recovery [88]. Despite legislation in Indonesia specifying that farmed civets must be released after five years of coffee production, the same legislation encourages the release of civets into the civet pet trade [94]. After five years in these facilities, few civets would be likely to survive in the wild due to psychosis and injury. However, the transfer of civets from coffee farms into the pet industry only continues their exploitation. Upon retirement from civet coffee farms, civets are usually too aggressive and their physical condition too unappealing to be rehomed as companion animals. Thus, individuals may find themselves used as breeding stock instead. Preliminary research into the civet pet trade illustrates comparable conditions between pet and civet coffee-farming facilities [27].

### 4.2. Civet Coffee and Civet Conservation

While it is illegal in many civet coffee-producing countries to harvest *Viverrid* species protected by international conservation agreements (such as binturong, which are listed as ‘vulnerable’ and Owston’s civet, which are listed as ‘endangered’ by the IUCN), these protections are poorly enforced in practise [86] and the indiscriminate capture methods (particularly snaring, which is endemic throughout Vietnam, Willcox et al. [95], puts all civet species (and other wildlife) at risk of physical and psychological harm. While conservation statuses are necessary to safeguard the most vulnerable species from unsustainable exploitation, conservation-based protections do not attribute value to the intrinsic interests of the individual and so overlook the relationship between individual animal welfare and the population health of a species. The social and ecological impacts of the overharvesting of civets are not well understood, nor is it clear how the mass capture of common palm civets from the wild for civet coffee production is impacting species population health and sustainability [87].

The assignment of species conservation statuses can also have colonial implications. Species classification is often non-reflective of how native species are perceived and engaged with by locals. The change in the conservation status of the binturong from ‘Least Concern’ to ‘Vulnerable to Extinction’ in 2008, for example, resulted in many Indonesian civet coffee farmers facing fines for housing binturong captive alongside common palm civets. As described in the ethnographic investigation of civet coffee production by Cahill [89,96], it is customary practise in Indonesia for civets of all species to be identified as one umbrella ‘musang’ or ‘luwak’ (‘civet’) species. Thus, where international agreements between countries recognise the difference between civet species and conservation needs, for many local people in range countries, these differences are seen as variations in civet morphology. The overnight change in legislation resulted in many civet coffee farmers receiving fines for holding binturong captive, and as word spread of the surprise inspections, many more farmers began releasing binturong from their collections [89]. While the release of wild animals may at first appear positive, there is no evidence to say that these animals were released in or near the locations where they were originally captured, and any injuries or behavioural abnormalities obtained in captivity could hinder their chances of wild survival. Thus, the welfare of these animals could have remained compromised even through their release, a phenomenon that resulted from the mismanagement of the species newly acquired conservation status. Thus, we see again how colonialism, conservation, and animal welfare are often entangled.

### 4.3. Colonisation of Animal Welfare in Civet Coffee Tourism

Alongside civet coffee’s global fame has come a rise in civet coffee tourism. Most prominent on the popular tourist destination island of Bali, civet coffee tours facilitate tourists desire to learn about the coffee’s unusual production process [26]. Tourists are guided through curated displays of coffee cherries, caged civets, civet faeces, and elderly Balinese women roasting coffee beans on an open fire. The short tour ends with a flute of endemic teas and coffees, typically served with panoramic views of the Balinese jungle and traditional rice paddies. In my previous review of 3364 Trip Advisor reviews (reported fully in [26]), I found that where some tourists claimed their civet coffee purchasing decisions were driven by curiosity or the desire to take part in a perceived ‘once in a lifetime experience’, others expressed a desire to support what they believed to be an authentic agricultural establishment and traditional practises. However, civet coffee tours are curated enterprises designed to exploit the colonial narrative of civet coffee’s origins, thus fulfilling the tourists’ expectations of traditional Balinese agri-food practises [97]. In such scenarios, animal welfare becomes secondary to tourist curiosity, with caged civets experiencing similar conditions to those in the intensive civet coffee production facilities.

Carder et al. [90] investigated 16 Indonesian civet coffee tourism sites and revealed significant animal welfare concerns present across survey locations. Display civets in tourist sites were subjected to poor housing and husbandry provisions, none of which were adequate in meeting basic animal needs as outlined by the Five Freedoms. No provisions for enrichment were observed; all civets had either limited or no access to clean water, and the animals were forced to stand and sleep on either wire mesh or slatted floors, conditions similar to those that have been reported for civet production farms [86,88]. Carder et al. [90] also noted that tour guides openly confirmed the civet’s inclusion was for display purposes only, whereas civet coffee production took place in locations non-disclosed to tourists. The secretive nature of civet coffee farming was likened by [89] to [98] ‘out-of-the-way places’, spaces that are free from the judgement of onlookers. Of course, civets in tourist and production facilities are intimately connected.

Without the demand for civet coffee, there would not be a heightened demand for civet capture, mass farming, or tourism. However, the relationship between consumer behaviour and issues of animal welfare and conservation is not always well understood by visitors to tourist facilities that house living animals. In fact, visitors to animal-based tourism (including zoos and other wildlife interaction experiences) overwhelmingly fail to recognise the negative implications of the experience for animals. The analysis of Moorehouse et al. (2015) of tourist perceptions of wildlife-tourist attractions found only 7.8% of tourists recognised signs of poor animal welfare or negative conservation impacts. My research into civet coffee tourism found similarly low levels. In total, 11.65% of English-speaking national and international tourists raised animal welfare concerns, with only 1.16% noting the possible conservation implications, all of whom had visited facilities where wild-caught civets were either caged or sedated to enable cage-free photographs [26].

Zoos in economically advanced nations can be used by tourists as a mechanism to either condone or condemn the animal welfare implications of civet coffee tours. For the latter, zoos are used by tourists as vehicles of judgement towards the Balinese tour guides for providing captive conditions less suitable for promoting animal welfare than would be experienced by animals in zoos within economically advanced regions [26]. The recognition that captive animal welfare can differ between animal tourism facilities shows that modern zoos have been somewhat successful at ‘bringing in’ humans to what visitors largely perceive as animal-dominated landscapes [99], even though most zoos in economically advanced regions of the world occur in urban spaces [32]. The rebranding of zoo ‘specimens’ as ‘ambassadors’ combined with immersive naturalistic ‘enclosures’ or ‘habitats’ rather than ‘cages’ [100] further acts as a semantic dilution of the historic colonial rhetoric of zoos, and so tourists do not always question the ethics of keeping animals captive. Although advances in zoo infrastructure have improved animal welfare in many zoos across the world, the co-opting of animal welfare as a mechanism for cultural judgement simply highlights the ongoing issue of colonialism within the tourism industry. In such cases, tourists overlook the economic requirements of high welfare practises and, in their judgements, are insensitive to the colonial rhetoric that comes with assumptions of animal cruelty as inherent to a particular culture. Most notably, such commentary is indicative of a lack of reflexivity from tourists who do not always recognise their role as the primary target audience. This issue thus illustrates the need for greater efforts to be made by contemporary zoos in economically advanced nations to be transparent with their visitors about their industries history, the interplay between tourism and colonialism, and how tourists themselves can contribute to positive animal welfare within the tourism landscape.

My research has also shown, however, that accredited zoos can be used by tourists as a way to overlook the welfare implications of the civets’ captive conditions [26]. In such cases, tourists will claim to see no ethical difference between Balinese civet coffee tours and accredited zoos, as both hold animals’ captive for display purposes. Thus, for these tourists, animal welfare is not a priority consideration for captive management. The perceived societal acceptance of animals used for public display (granted, this is but one objective of the modern zoo environment) overlooks both the complexities involved in providing positive captive animal welfare and the wider conservation impact of unregulated wild capture that is synonymous with the civet coffee industry. In highlighting the utility of the animal’s captivity, tourists attempt to negate cognitive dissonance caused by the possible harms incurred by the animals’ capture and display. Where tourists commented on there being little difference between animals housed on display in coffee tours and those on display in zoos (see [26]), questions must be raised as to the impact of how modern zoos portray their animals to visitors and the wider public. Indeed, close human-animal encounters are effective marketing tools. Of the tourists who reported being able to pet, hold, feed, or have photographs taken with civets, many went on to recommend the experience to others [26].

In their attempts to meet their educational aims, accredited zoos can muddy the boundaries between education, recreation, and conservation [61]. Inappropriate portrayals of zoo animals can ultimately lead to zoo visitors (both those who visit in person and those who engage with online entertainment-driven marketing campaigns [101]), developing a poor understanding of captive animal welfare. Thus, zoos risk contributing to the endorsement of unethical, unregulated animal-based tourism. Given the frequency at which animal-visitor interactions are facilitated by accredited zoos the world over [62], it is therefore somewhat unsurprising that few tourists consider the negative implications of feeding animals when abroad, whether in free-ranging, semi-captive, or captive settings [102]. Wildlife selfies are equally problematic for wildlife in tourism [103], and tourists may not be able to recognise the animal welfare or conservation implications of paying a fee to pose with wild animals when similar close contact experiences are available from accredited zoos in their home countries. Accrediting bodies have written guidelines for their members when offering animal-visitor interactions, which include measures to ensure that animal welfare and conservation messaging are upheld through the encounter [104]. However, not all messaging is effectively obtained by the public, either when delivered to the visitor partaking in the interaction [105,106] or via marketing media that can lack educational context [107,108]. Given the predicted intensification of demand for human-animal encounters in tourism [36], accredited zoos ought to be cognizant of their formative role as educators for the promotion of positive animal welfare and conservation within global tourism.

## 5. Towards Decolonisation of Animal Welfare in Tourism

While it is not possible for zoos to completely decolonise, given that the fundamental attribute of zoos is to care for animals in a captive setting, zoos could offer visitors steppingstones towards more ethical human-wildlife encounters within the remit of tourism and, more generally, as consumers within capitalist systems. Indeed, as noted by [63], in relation to the issue of zoos continuing to offer a merger between entertainment and education, “old views are not simply consigned to the dustbin of history when new ones emerge. Instead, they tend to linger, overlapping with newly emergent ideas”. With 700 million people said to visit accredited zoos globally each year [38], contemporary zoos are in a powerful position to advocate for greater reflexivity to be adopted by tourists and the tourism industry concerning the treatment of animals.

First, however, the accredited zoo industry must recognise and reflect on current practices that overlap with the industries’ colonial origins; origins that accredited zoos have made substantial strides to move beyond. For one, the ways accredited zoos engage visitors with animals (particularly the feeding and petting of wild animals) ought to be re-evaluated to ensure tourists are clear on the distinctions between high- and low-welfare animal attractions when faced with similar opportunities in unregulated facilities. Zoos could also offer more tangible ways for visitors to reflect on their behaviour and their impact on species protection by building on previously successful ethical consumerism campaigns. Zoo visitors are more likely to adopt pro-conservation behaviour during their visit than after [42], and some zoos have reported highly successful engagement rates when bringing ethical consumption into the zoo space. Smartphone recycling stations at gorilla exhibits to educate visitors about coltan mining [109] and sustainable palm oil educational initiatives at orangutan exhibits [78] have shown innovation within the zoo industry towards effective education concerning the topics of sustainability and ethical consumerism. However, these initiatives are not synonymously adopted throughout accredited zoo facilities, and many species (including civets) could contribute to these initiatives. Thus, a more nuanced species selection process could be adopted. By doing so, finite resources could be better optimised in terms of achieving more of the zoo industries contemporary objectives while also addressing the overrepresentation of endangered or otherwise charismatic species [69,110].

The first suggestion I make, therefore, towards the decolonisation of animal welfare and conservation within animal tourism is for the selection of zoo species to be made based upon a holistic assessment of the species’ ability to meet education, conservation, and animal welfare objectives. The repatriation of endangered species to their countries of origin should therefore occur where either: the species in question is unlikely to be returned to the wild from their present location outside of their range country; the captive population is too small or genetically unsustainable to be managed in captivity without the further introduction of wild-captured individuals; insufficient funds are being redirected from the holding zoo to in situ conservation initiatives; or the dominant conservation threat can be more effectively addressed by engaging local communities in the species native range countries. Thus, while not all endangered species would be phased out of economically advanced zoos, the zoo industry would be increasingly accountable for their contribution to the species’ protection while optimising the limited resources available to them.

In part to address possible concerns related to the disparate animal welfare conditions between zoos in economically advanced and emerging economic regions, where some of these species would be returned, programmes should be established between the current holding collections in colonial nations and accredited zoos in range of countries. These programmes should serve to support non-accredited zoos throughout the species range to deliver higher standards of animal welfare and education so that they can achieve accreditation. Thus, a global cooperative of zoos should contribute to sustainable tourism development [111]. In doing so, zoos would better serve the interests of animal welfare and conservation as a global animal tourism network, thus reaching more animals and more human visitors across the world. A unified global approach to the issue of animal welfare in tourism is needed, as there is currently no global governing body for the various forms of wildlife tourism attractions on offer [112]. While it is not to say that all modern zoos are failing to support in situ initiatives or falling short of their animal welfare obligations, it must be recognised that not all accredited zoos are fulfilling their potential in meeting these objectives [113]. Nor do zoos adequately respond to the issue of poor animal welfare within global tourism. A more holistic framework for collection planning in cooperation with zoos in species home ranges would thus assist zoos globally in meeting such objectives while decolonising animal tourism from within.

To ensure the welfare of animals during repatriation efforts, I recommend a phased programme be implemented for individuals whose welfare may be compromised by travelling or whose survivability might be reduced by their transfer. For example, this approach would apply if the animal were elderly, unwell, or where there was an insufficiency of captive facilities available in their range country that could maintain comparatively high levels of welfare. In place of the species removed from zoos within non-range nations, their non-endangered proxy relatives should be housed instead (either once the original individual has died, been retired from the public-facing collection, or after repatriation). Thus, zoos would begin transitioning to a proxy species approach, a form of surrogate defined as “a non-threatened species that are morphologically similar [to an endangered species] but not otherwise represented in zoos” [69]. A proxy approach would allow more zoo animals to fulfil the role of conservation ambassadors while keeping the rarest species within their range countries. I would, however, extend the definition of proxy species to include the representation of species either already formally recognised as endangered, those in steady decline, or those otherwise threatened by poor welfare practises. Thus, proxy species could provide both conservation and animal welfare education messages.

A greater representation of common palm civets in zoos within economically advanced regions would also offer further opportunities to educate zoo visitors about their role in global animal welfare issues. The lack of common palm civets in zoos in economically advanced nations means the only place to see common palm civets and learn about civet coffee in-person is through visiting locations in Asia where civets are extracted from the wild and housed in conditions with proven welfare implications [88,90]. The display of common palm civets in zoos in economically advanced regions could decrease the allure of the species exoticism associated with civet coffee. Education messages concerning the role of consumerism in global animal welfare and conservation issues would provide visitors with tangible ways to contribute towards sustainability through ethical consumer practises. Indeed, zoo visitors have reported a lack of tangible actions as a barrier to adopting pro-conservation behaviour beyond donating money [114]. Furthermore, the broadening of educational messages in zoos to include global animal welfare concerns would optimise the educational potential of ambassador animals. Of course, in the case of civets, this would continue to require zoos to carefully consider how the species is portrayed so as not to increase the novelty appeal of civet coffee or to promote close-contact interactions, which could be harmful to zoo residents and other animals in tourism.

My second suggestion for the decolonisation of animal tourism, therefore, is for zoos in economically advanced nations to reflect on their portrayals of wild animals to their zoo visitors. The growing interest in the relationships held between humans and animals in tourism [114,115,116,117,118], and the shift towards what Caton [119] calls the “moral turn” in tourism scholarship towards research that advocates for sustainable tourism, is indicative of a theoretical shift toward a species turn already underway in Anthropological practise [25]. As long-standing features of human societies and icons of animal tourism across the globe, modern zoos must respond to the call to enhance the welfare of animals in the tourism landscape more broadly than within the confines of the zoo walls. As of yet, the conservation and animal welfare messages presented to tourists have been inconsistently delivered as zoos increasingly rely on close contact with animals experience, such as petting, feeding, and posing with wild animals, to attract zoo visitors [62]. Should zoos be considered of paramount importance for the conservation of species and the pro-conservation education of the public, then zoos across the world require additional support to meet these objectives. Indeed, in the age of the Anthropocene, the sixth mass extinction largely due to humans [120], the role of zoos in global conservation ought not to be understated [113]. If zoo institutions were less dependent on revenue generated by zoo visitors, zoos would have less need to monetise their animal residents. Thus, the commodity value of zoo animals could fully progress from entertainer to conservation and animal welfare education ambassador. To this end, national governments and local authorities must support zoos financially, recognising the increasing need to respond to the current ecological crisis.

Finally, I argue that contemporary zoos in high-income nations ought to be transparent with their visitors about the advances made for animal welfare and conservation since the industries’ emergence in the colonial period. While the repatriation of endangered species to their native countries and the cessation of animal encounters and performances would aid with this objective, for full educational benefits, zoo visitors should be informed about the progressively evolving nature of animal welfare and conservation science. Of particular focus should be the role of the public in demanding higher welfare standards for zoo animals, to encourage personal reflexivity towards ethical consumer and tourist behaviour.

## 6. Conclusions

In this paper, I have brought two forms of animal commodification into dialogue, namely the management of civets in zoos and the rising trend in civet coffee production and tourism. Through an analysis of data held on the global database ‘Zoological Information Management Systems’ (ZIMS), I have shown that charismatic, rare, and endangered *Viverrids* are most commonly represented in zoos within economically advanced regions of the world, far from the species natural range countries. ZIMS records also showed that common species, such as the common palm civet, were rarely present in zoos in economically advanced non-range countries, despite the numerous educational benefits the species could provide to zoo visitors. Although the IUCN lists the Common palm civet as a species of Least Concern [121], the civet coffee industry threatens the species both in terms of its conservation and individual welfare. The Common palm civets are enrolled as coffee producers in intensive low-welfare farms and as display animals in civet coffee tourism sites across Asia, industries fuelled by an ever-growing global consumer demand. Thus, common palm civets could educate zoo visitors about the conservation and welfare implications of the civet coffee industry and provide zoo visitors with tangible solutions to these and similar species issues through sustainable consumerism and tourism.

Further analysis of tourist perceptions of civet coffee tourism in Bali revealed the interplay between accredited zoos and unregulated, low-welfare, animal-based attractions. Tourists would either use zoos to justify or condemn civet coffee tourism, and many saw little difference between unregulated facilities and accredited zoos, as both offer a range of close-contact experiences with wild animals. A common theme across tourist perceptions of civet coffee tourism was a colonial narrative that is harmful to both humans and animals. These findings, therefore, highlight the need for further efforts to be made to decolonise global animal tourism. By critiquing the ways that some accredited zoo practises can be reflective of the industry’s colonial past, I have argued that accredited zoos are well positioned within the global tourism landscape to lead efforts towards the decolonisation of animal tourism. Indeed, the modern zoo has already made significant strides in moving past its colonial origins.

In all, I make several theoretical suggestions on how zoos could move towards the decolonisation of animal tourism from within. My suggestions include the careful repatriation of rare species to their home countries and the use of proxy species to educate tourists about ethical consumerism and tourism. The animal welfare considerations of repatriation are discussed, along with the need for zoos in economically advanced nations to work collectively with zoos in the range of countries of repatriated animals to ensure high levels of welfare, conservation, and education are upheld on a global scale. In the immediate future, I ask the zoo industry to critically evaluate the current applications of zoo animals as educational entertainers. The cessation of certain animal-visitor interactions in accredited zoos (such as petting and feeding wild animals) would set a clearer example for tourists so they could more easily recognise the distinctions between high and low-welfare animal tourism. Finally, I propose zoos offer greater transparency to their visitors about the progress already made since the zoo industry’s colonial emergence.

## Figures and Tables

**Table 1:** Number of Viverrids housed in zoos recorded on the Zoological Information Management Systems (ZIMS) database as of 2023 [70]. (Table 1 comprised 365 holding collections across the globe that house *Viverrids* and use the ZIMS database to record their species data. Of these holding collections, 84% (306) were accredited by WAZA and/or regional or national associations, and only 16% (59) were unaccredited. Thus, data are lacking for unaccredited facilities, and so Table 1 represents a conservative estimate of *Viverrids* housed as part of global zoo tourism. While not all zoos across the globe use ZIMS for storing their records, the ZIMS database is the most comprehensive zoo database in the world (see https://species360.org/, accessed on 18 April 2023). Therefore, at the time of writing, the data presented here present the most accurate picture of *Viverrid* holdings currently available. The monetary total has been converted from Euros to US dollars for consistency).

Species	IUCN Status	Emerging Economies	Advanced Economies	Total
African civet, *Civettictis civetta*	LC	5	15	20
Banded linsang, *Prionodon linsang*	LC	5	0	5
Banded palm civet, *Hemingalus derbyanus*	VU	3	10	13
Binturong, *Arctictis binturong*	VU	199	288	487
Common palm civet, *Paradoxurus hermaphroditus*	LC	124	24	148
Forest Genet, *Genetta vistoriae*	LC	0	6	6
Genet (*species undocumented*)	LC	12	4	16
Golden palm civet, *Paradoxurus zeylonesis*	VU	9	0	9
Hausa genet, *Genetta thierryi*	LC	0	2	2
Large Indian civet, *Viverra zibetha*	LC	19	0	19
Large-spotted civet, *Viverra magaspila*	EN	5	0	5
Large-spotted genet, *Genetta pardina*	LC	20	19	39
Masked palm civet, *Paradoxurus larvata*	LC	36	51	87
Owston’s palm civet, *Chrotogale owstoni*	EN	16	9	25
Small Indian civet, *Viverricula indica*	LC	29	1	30
Small-spotted genet, *Genetta genetta*	LC	38	41	79
Small-toothed palm civet, *Arctogalidia trivirgata*	LC	16	15	31
Spotted linsang, *Prionodon pardicolor*	LC	1	0	1
TOTAL		537	485	1022

## References

[1] Carr N. (2016). Star Attractions and Damp Squibs at the Zoo: A Study of Visitor Attention and Animal Attractiveness. Tour. Recreat. Res..

[2] Veron G., Debruille A., Kayser P., Fernandez D.A.P., Bourgeois A. (2020). Genetic Diversity and Structure of the Binturong *Arctictis binturong* (Carnivora: Viverridae)-Status of the Elusive Palawan Binturong and Implications for Conservation. Zool. J. Linn. Soc..

[3] Bourgeois A., Kayser P., Debruille A., Veron G. (2020). Binturong *Arctictis binturong* Conservation: The Relationship between the Zoo Community and ABConservation for an Integrated Conservation Programme in Palawan, Philippines. Int. Zoo Yearb..

[4] Finch K., Leary M., Holmes L., Williams L.J. (2022). Zoo Closure Does Not Affect Behavior and Activity Patterns of Palawan Binturong (*Arctictis binturong whitei*). J. Zool. Bot. Gard..

[5] Greene L.K., Wallen T.W., Moresco A., Goodwin T.E., Drea C.M. (2016). Reproductive Endocrine Patterns and Volatile Urinary Compounds of *Arctictis binturong*: Discovering Why Bearcats Smell like Popcorn. Sci. Nat..

[6] Glachet O., Moustafa A.A., Gallouj K., El Haj M. (2019). Smell Your Memories: Positive Effect of Odor Exposure on Recent and Remote Autobiographical Memories in Alzheimer’s Disease. J. Clin. Exp. Neuropsychol..

[7] Willcox D.H.A., Chutipong W., Gray T.N.E., Cheyne S., Semiadi G., Rahman H., Coudrat C.N.Z., Jennings A., Ghimirey Y., Ross J. (2016). *Arctictis* *binturong*. IUCN Red List Threat. Species.

[8] Carr N. (2016). Ideal Animals and Animal Traits for Zoos: General Public Perspectives. Tour. Manag..

[9] Hooper J., Linna M., Kassinen S., Salvage J. (2022). Technologies, Bodies and Faecal Matters: Embodied Empathy with Coffee Producing Civets. Transpositiones J. Transdiscipl. Intermedial Cult. Stud..

[10] Roe K., McConney A., Mansfield C.F. (2014). The Role of Zoos in Modern Society—A Comparison of Zoos’ Reported Priorities and What Visitors Believe They Should Be. Anthrozoos.

[11] Falk J.H., Reinhard E.M. (2007). Why Zoos & Aquariums Matter: Assessing the Impact of a Visit to a Zoo or Aquarium.

[12] Marino L., Lilienfeld S.O., Malamud R., Nobis N., Broglio R. (2010). Do Zoos and Aquariums Promote Attitude Change in Visitors? A Critical Evaluation of the American Zoo and Aquarium Study. Soc. Anim..

[13] Falk J.H., Heimlich J.E., Vernon C.L., Bronnenkant K. (2010). Critique of a Critique: Do Zoos and Aquariums Promote Attitude Change in Visitors?. Soc. Anim..

[14] Malamud R., Kalof L. (2017). The Problem with Zoos. The Oxford Handbook of Animal Studies.

[15] Grajal A., Luebke J.F., Kelly L.A.D.G., Matiasek J., Clayton S., Karazsia B.T., Saunders C.D., Goldman S.R., Mann M.E., Stanoss R. (2017). The Complex Relationship between Personal Sense of Connection to Animals and Self-Reported Proenvironmental Behaviors by Zoo Visitors. Conserv. Biol..

[16] Clarke A.G. (2009). The Frozen Ark Project: The Role of Zoos and Aquariums in Preserving the Genetic Material of Threatened Animals. Int. Zoo Yearb..

[17] Gurian E. (2005). Threshold Fear. Reshaping Museum Space.

[18] Dick G. (2017). Natural History Museums, Zoos, and Aquariums. The Future of Natural History Museums.

[19] Anderson K. (2018). Culture and Nature at the Adelaide Zoo: At the Frontiers of ‘Human’ Geography. Environment.

[20] Zelenka J., Paskova M. (2002). Výkladový Slovník Cestovního Ruchu.

[21] Vaníček J. (2012). Comparison of Visitors’ Profile in Selected Zoological Gardens in the Czech Republic. COT Bus..

[22] Nekolný L., Fialová D. (2018). Zoo Tourism: What Actually Is a Zoo?. Czech J. Tour..

[23] Szczygielska M. (2020). Elephant Empire: Zoos and Colonial Encounters in Eastern Europe. Cult. Stud..

[24] Pratt M.L. (1992). Imperial Eyes: Travel Writing and Transculturation.

[25] Kirksey S.E., Helmreich S. (2010). The Emergence of Multispecies Ethnography. Cult. Anthropol..

[26] Hooper J. (2022). Cat-Poo-Chino and Captive Wildlife: Tourist Perceptions of Balinese Kopi Luwak Agrotourism. Soc. Anim..

[27] Hooper J. (2022). Contamination: The Case of Civets, Companionship, COVID, and SARS. J. Appl. Anim. Welf. Sci..

[28] Song Z. (2021). Between the Past and Future. Antwerp Zoo and the 19th Century Belgium. Tech. Soc. Sci. J..

[29] Sampaio M.B., Schiel N., Souto A. (2020). From Exploitation to Conservation: A Historical Analysis of Zoos and Their Functions in Human Societies. Ethnobiol. Conserv..

[30] Coleman D. (2013). Animal Studies Journal Menageries and Museums: John Simons’ The Tiger That Swallowed the Boy (2012) and the Lives and Afterlives of Historical Animals. Anim. Stud. J..

[31] Abbattista G. (2015). Beyond the ‘Human Zoos’ This Contribution Discusses Some Aspects of What Is Conventionally Termed as ‘Living Human’ or ‘Ethnic Exhibitions’: An Expression Denoting the Mainly Nineteenth-Early Twentieth Century Western Practice of Putting on Display.

[32] Braverman I. (2012). Zooland: The Institution of Captivity.

[33] Rothfels N. (2002). Savages and Beasts: The Birth of the Modern Zoo.

[34] Hosey G., Melfi V., Pankhurst S. (2009). Zoo Animals: Behaviour, Management and Welfare.

[35] Dibley B., Hawkins G. (2019). Making Animals Public: Early Wildlife Television and the Emergence of Environmental Nationalism on the ABC. Continuum.

[36] Carr N., Broom D.M. (2018). Tourism and Animal Welfare.

[37] Mason G.J., Latham N. (2004). Can’t Stop, Won’t Stop: Is Stereotypy a Reliable Animal Welfare Indicator?. Anim. Welf..

[38] Moorhouse T.P., Dahlsjö C.A., Baker S.E., D’Cruze N.C., Macdonald D.W. (2015). The Customer Isn’t Always Right—Conservation and Animal Welfare Implications of the Increasing Demand for Wildlife Tourism. PLoS ONE.

[39] Warsaw D., Sayers J. (2020). The Influence of Animal Welfare Accreditation Programmes on Zoo Visitor Perceptions of the Welfare of Zoo Animals. Res. Artic. J. Zoo Aquarium Res..

[40] Altman J.D. (1998). Animal Activity and Visitor Learning at the Zoo. Anthrozoos.

[41] Godinez A.M., Fernandez E.J., Morrissey K. (2013). Visitor Behaviors and Perceptions of Jaguar Activities. Anthrozoos.

[42] Godinez A.M., Fernandez E.J. (2019). What Is the Zoo Experience? How Zoos Impact a Visitor’s Behaviors, Perceptions, and Conservation Efforts. Front. Psychol..

[43] Miller L.J. (2012). Visitor Reaction to Pacing Behavior: Influence on the Perception of Animal Care and Interest in Supporting Zoological Institutions. Zoo Biol..

[44] EAZA EAZA Standards for the Accommodation and Care of Animals in Zoos and Aquaria. European Associastion of Zoos and Aquaria. 2019; p. 19. http://www.eaza.net/assets/Uploads/Standards-and-policies/Standards-for-the-Accommodation-and-Care-of-Animals-2014.pdf.

[45] WAZA WAZA Code of Ethics and Animal Welfare. Proceedings of the 58th Annual Meeting, of the World Association of Zoos and Aquariums.

[46] Agoramoorthy G., Hsu M.J. (2005). Use of Nonhuman Primates in Entertainment in Southeast Asia. J. Appl. Anim. Welf. Sci..

[47] Primack R.B. (2002). Essentials of Conservation Biology.

[48] Collard R.C. (2013). Panda Politics. Can. Geogr..

[49] Meehan C.L., Mench J.A., Carlstead K., Hogan J.N. (2016). Determining Connections between the Daily Lives of Zoo Elephants and Their Welfare: An Epidemiological Approach. PLoS ONE.

[50] Chrulew M., Rose D.B., van Dooren T., Chrulew M. (2017). Saving the Golden Lion Tamarin. Extinction Studies: Stories of Time, Death and Generations.

[51] Zhang Y., Wang F., Cui Z., Li M., Li X., Ye X., Yu X. (2021). Can We Reestablish a Self-Sustaining Population? A Case Study on Reintroduced Crested Ibis with Population Viability Analysis. Avian Res..

[52] Pukazhenthi B.S., Wildt D.E. (2004). Which Reproductive Technologies Are Most Relevant to Studying, Managing and Conserving Wildlife?. Reprod. Fertil. Dev..

[53] Biggins D.E., Hanebury L.R., Miller B.J., Powell R.A. (2011). Black-Footed Ferrets and Siberian Polecats as Ecological Surrogates and Ecological Equivalents. J. Mammal..

[54] Martin-Wintle M.S., Shepherdson D., Zhang G., Zhang H., Li D., Zhou X., Li R., Swaisgood R.R. (2015). Free Mate Choice Enhances Conservation Breeding in the Endangered Giant Panda. Nat. Commun..

[55] Masilkova M., Boukal D., Ash H., Buchanan-Smith H.M., Konečná M. (2022). Linking Personality Traits and Reproductive Success in Common Marmoset (*Callithrix jacchus*). Sci. Rep..

[56] Blais B.R., Wells S.A., Poynter B.M., Koprowski J.L., Garner M.M., Allard R.A. (2022). Adaptive Management in a Conservation Breeding Program: Mimicking Habitat Complexities Facilitates Reproductive Success in Narrow-Headed Gartersnakes (*Thamnophis rufipunctatus*). Zoo Biol..

[57] Ludwig C., Dehnhard M., Pribbenow S., Silinski-Mehr S., Hofer H., Wachter2 B. (2019). Asymmetric Reproductive Aging in Cheetah (*Acinonyx jubatus*) Females in European Zoos. J. Zoo Aquarium Res..

[58] Farquharson K.A., Hogg C.J., Grueber C.E. (2018). A Meta-Analysis of Birth-Origin Effects on Reproduction in Diverse Captive Environments. Nat. Commun..

[59] Guenther K.M. (2020). The Lives and Deaths of Shelter Animals: The Lives and Deaths of Shelter Animals.

[60] Greggor A.L., Vicino G.A., Swaisgood R.R., Fidgett A., Brenner D., Kinney M.E., Farabaugh S., Masuda B., Lamberski N. (2018). Animal Welfare in Conservation Breeding: Applications and Challenges. Front. Vet. Sci..

[61] Fernandez E.J., Tamborski M.A., Pickens S.R., Timberlake W. (2009). Animal-Visitor Interactions in the Modern Zoo: Conflicts and Interventions. Appl. Anim. Behav. Sci..

[62] D’Cruze N., Khan S., Carder G., Megson D., Coulthard E., Norrey J., Groves G. (2019). A Global Review of Animal–Visitor Interactions in Modern Zoos and Aquariums and Their Implications for Wild Animal Welfare. Animals.

[63] Carr N. (2011). 5 Zoos and Animal Encounters to Touch or Not to Touch, That Is the Question. Wild Animals and Liesure: Rights and Welfare.

[64] EAZA Policy and Legislation. https://www.eaza.net/about-us/areas-of-activity/policyandlegislation/.

[65] Jepson P., Barua M. (2015). A Theory of Flagship Species Action. Conserv. Soc..

[66] Favreau J.M., Drew C.A., Hess G.R., Rubino M.J., Koch F.H., Eschelbach K.A. (2006). Recommendations for Assessing the Effectiveness of Surrogate Species Approaches. Biodivers. Conserv..

[67] Branton M., Richardson J.S. (2011). Assessing the Value of the Umbrella-Species Conceptfor Conservation Planning with Meta-Analysis. Conserv. Biol..

[68] Macdonald E.A., Hinks A., Weiss D.J., Dickman A., Burnham D., Sandom C.J., Malhi Y., Macdonald D.W. (2017). Identifying Ambassador Species for Conservation Marketing. Glob. Ecol. Conserv..

[69] Kerr K.C.R. (2021). Zoo Animals as “Proxy Species” for Threatened Sister Taxa: Defining a Novel Form of Species Surrogacy. Zoo Biol..

[70] ZIMS Species Holdings Species360 Zoological Information Management System. https://species360.org/.

[71] Whitehouse-Tedd K., Spooner S., Scott L., Lozano-Martinez J., Berger M., Corbett S. (2018). Animal Ambassador Encounter Programmes in Zoos: Current Status and Future Research Needs. Zoo Animals: Husbandry, Welfare and Public Interactions. Animal Science, Issues and Research.

[72] Spooner S.L., Farnworth M.J., Ward S.J., Whitehouse-Tedd K.M. (2021). Conservation Education: Are Zoo Animals Effective Ambassadors and Is There Any Cost to Their Welfare?. J. Zool. Bot. Gard..

[73] Gusset M., Dick G. (2011). The Global Reach of Zoos and Aquariums in Visitor Numbers and Conservation Expenditures. Zoo Biol..

[74] Barongi R., Fisken F.A., Parker M., Gusset M. (2015). Committing to Conservation: The World Zoo and Aquarium Conservation Strategy.

[75] AZA (2021). 2020 Annual Report on Conservation and Science. www.aza.org/annual-report-on-conservation-and-science.

[76] Gusset M., Dick G. (2010). ‘Building a Future for Wildlife’? Evaluating the Contribution of the World Zoo and Aquarium Community to in Situ Conservation. Int. Zoo Yearb..

[77] Research S. Kopi Luwak Coffee Market Size Is Projected to Reach USD 10 Billion by 2030, Growing at a CAGR of 4.9%: Straits Research. Global Newswire. https://www.globenewswire.com/en/news-release/2022/10/04/2528177/0/en/Kopi-Luwak-Coffee-Market-Size-is-projected-to-reach-USD-10-Billion-by-2030-growing-at-a-CAGR-of-4-9-Straits-Research.html.

[78] Pearson E.L., Lowry R., Dorrian J., Litchfield C.A. (2014). Evaluating the Conservation Impact of an Innovative Zoo-Based Educational Campaign: “Don’t Palm Us Off” for Orang-Utan Conservation. Zoo Biol..

[79] Hooper J. (2023). Civets in Society: What Can the Family Viverridae Teach Us about Disappearance in the Anthropocene.

[80] Marcone M.F. (2004). Composition and Properties of Indonesian Palm Civet Coffee (Kopi Luwak) and Ethiopian Civet Coffee. Food Res. Int..

[81] Bickley J. The History of Kopi Luwak. Gayo Kopi. https://gayokopi.com/history-of-kopi-luwak/?unapproved=54473&moderation-hash=0c64ec7b218a3bd26ae847c8c30ee324#comment-54473.

[82] Nakashima Y., Inoue E., Inoue-Murayama M., Sukor J.R.A. (2010). Functional Uniqueness of a Small Carnivore as Seed Dispersal Agents: A Case Study of the Common Palm Civets in the Tabin Wildlife Reserve, Sabah, Malaysia. Oecologia.

[83] (2020). Kopi Luwak Direct. Shop Kopi Luwak Direct [Online]. https://kopiluwakdirect.com/kopi-luwak-taste/.

[84] Muzaifa M., Hasni D., Rahmi F. (2019). What Is Kopi Luwak? A Literature Review on Production. Earth and Environmental Science.

[85] Ifmalinda I., Setiasih I.S., Muhaemin M., Nurjanah S. (2019). Chemical Characteristics Comparison of Palm Civet Coffee (Kopi Luwak) and Arabica Coffee Beans. J. Appl. Agric. Sci. Technol..

[86] Thi M.T., Gray R., Pham T., Thuy H.T., Le Kim L., Nhat L.C., Nguyen T. Commercial Civet Farming Practices and Conservation Impacts on Wild Civet Populations in Central Vietnam. 2022; pp. 1–27. https://assets.researchsquare.com/files/rs-1806075/v1/0d558491-d773-48ae-9ae5-602b2d258753.pdf?c=1658227465.

[87] Shepherd C.R. (2012). Observations of Small Carnivores in Jakarta Wildlife Markets, Indonesia, with Notes on Trade in Javan Ferret Badger Melogale Orientalis and on the Increasing Demand for Common Palm Civet Paradoxurus Hermaphroditus for Civet Coffee Prod. Small Carniv. Conserv..

[88] Lynn G., Rogers C. (2013). Civet Cat Coffee’s Animal Cruelty Secrets. https://www.bbc.co.uk/news/uk-england-london-24034029.

[89] Cahill C.W. (2017). Feral Natures and Excremental Commodities: Purity, Scale, and the More-than-Human in Indonesia.

[90] Carder G., Proctor H., Schmidt-Burbach J., D’cruze N. (2016). The Animal Welfare Implications of Civet Coffee Tourism in Bali. Anim. Welf..

[91] Lewis E., Boyle L.A., O’Doherty J.V., Brophy P., Lynch P.B. (2005). The Effect of Floor Type in Farrowing Crates on Piglet Welfare. Irish J. Agric. Food Res..

[92] Hunter L. (2019). Carnivores of the World.

[93] World Animal Protection (2020). Civet Coffee: Campaigning for Cage-Free. https://www.worldanimalprotection.org.uk/campaigns/animals-wild/civet-coffee-kopi-luwak.

[94] Peraturan Menteri Pertanian Permentan Tentang Cara Produksi Kopi Luwak Melalui Pemeliharaan Luwak Yang Memenuhi Prinsip Kesejahteraan Hewan Nomor 37 Tahun 2015. https://peraturan.bpk.go.id/Home/Details/160544/permentan-no-37permentankb12062015-tahun-2015.

[95] Willcox D., Taylor O., Jimerson J., Underwood G., Nguyen V. (2020). Owston’s Civet *Chrotogale owstoni*: A Priority for Conservation Breeding. Int. Zoo Yearb..

[96] Cahill C. (2020). [Un]becoming a Resource: Translating the Nature of Civets in Indonesia. Ethnos.

[97] Hooper J., Lopez A., Venegas Q., Kline C. (2023). Fill Your Cup: A Multpspecies Exploration of Balinese Kopi Luwak Agrotourism. Tourism, Heritage and Commodification of Non-Human Animals: A Posthumanist Reflection.

[98] Tsing A.L. (1993). In the Realm of the Diamond Queen: Marginality in an Out-of-the-Way Place.

[99] Grazian D. (2012). Where the Wild Things Aren’t: Exhibiting Nature in American Zoos. Sociol. Q..

[100] Pennisi L., Lackey N.Q., Holland S.M. (2017). Can an Immersion Exhibit Inspire Connection to Nature and Environmentally Responsible Behavior?. J. Interpret. Res..

[101] Carr N., Cohen S. (2011). The Public Face of Zoos: Images of Entertainment, Education and Conservation. Anthrozoos.

[102] Orams M.B. (2002). Feeding Wildlife as a Tourism Attraction: A Review of Issues and Impacts. Tour. Manag..

[103] Carder G., Plese T., Machado F.C., Paterson S., Matthews N., McAnea L., D’cruze N. (2018). The Impact of ‘Selfie’ Tourism on the Behaviour and Welfare of Brown-Throated Three-Toed Sloths. Animals.

[104] WAZA (2020). WAZA Guidelines for Animal-Visitor Interactions.

[105] Spooner S.L., Jensen E.A., Tracey L., Robert A. (2021). Evaluating the Effectiveness of Live Animal Shows at Delivering Information to Zoo Audiences Evaluating the Effectiveness of Live Animal Shows at Delivering Information to Zoo Audiences. Int. J. Sci. Educ..

[106] Whitehouse-tedd K.M., Lozano-martinez J., Reeves J., Martin J.H., Prozesky H., Lozano-martinez J., Reeves J. (2022). Assessing the Visitor and Animal Outcomes of a Zoo Encounter and Guided Tour Program with Ambassador Cheetahs. Anthrozoos.

[107] Llewellyn T., Rose P.E. (2021). Education Is Entertainment? Zoo Science Communication on YouTube. J. Zool. Bot. Gard..

[108] Shaw M.N., Mcleod E.M., Borrie W.T., Miller K.K. (2022). Human Positioning in Close-Encounter Photographs and the Effect on Public Perceptions of Zoo Animals. Animals.

[109] Litchfield C.A., Lowry R., Dorrian J. (2018). Recycling 115,369 Mobile Phones for Gorilla Conservation over a Six-Year Period (2009–2014) at Zoos Victoria: A Case Study of ‘Points of Influence’ and Mobile Phone Donations. PLoS ONE.

[110] Hosey G., Melfi V., Ward S.J., Francesco Maria Angelici L.R. (2020). Problematic Animals in the Zoo: The Issue of Charismatic Megafauna. Problematic Wildlife II: New Conservation and Management Challenges in the Human-Wildlife Interactions.

[111] WTO Sustainable Tourism Development. World Tourism Organization..

[112] Moorhouse T., D’Cruze N.C., Macdonald D.W. (2017). Unethical Use of Wildlife in Tourism: What’s the Problem, Who Is Responsible, and What Can Be Done?. J. Sustain. Tour..

[113] Miller B., Conway W., Reading R.P., Wemmer C., Wildt D., Kleiman D., Monfort S., Rabinowitz A., Armstrong B., Hutchins M. (2004). Evaluating the conservation mission of zoos, aquariums, botanical gardens, and natural history museums. Conserv. Biol..

[114] Nygren N.V., Ojalammi S. (2018). Conservation Education in Zoos—A Literature Review. TRACE.

[115] Fennell D. (2011). Tourism and Animal Ethics.

[116] Burns G.L. (2015). Animals as Tourism Objects: Ethically Refocusing Relationships Between Tourists and Wildlife. Anim. Tour. Underst. Divers. Relatsh..

[117] Winter C. (2020). A Review of Animal Ethics in Tourism: Launching the Annals of Tourism Research Curated Collection on Animal Ethics in Tourism. Ann. Tour. Res..

[118] Rickly J.M., Kline C. (2021). Introduction: Working for the (Hu)Man in the Tourism Industry. Exploring Non-Human Work in Tourism.

[119] Caton K. (2012). Taking the Moral Turn. Ann. Tour. Res..

[120] Crutzen P.J., Stoermer E.F. (2000). The “Anthropocene”. IGPB Glob. Chang. Newsl..

[121] Duckworth J.W., Timmins R.J., Choudhury A., Chutipong W., Willcox D.H.A., Mudappa D., Rahman H., Widmann P., Wilting A., Xu W. (2016). Paradoxurus hermaphroditus. IUCN Red List. Threat. Species.

